# Waning Humoral Immune Response Following the Third and Fourth SARS‐COV‐2 Vaccine: A Cohort Study in Healthcare Workers

**DOI:** 10.1111/irv.70003

**Published:** 2024-08-27

**Authors:** Ahmet Furkan Süner, Gül Ergör, Derya Çağlayan, Neslişah Türe, Irmak Güzel, Çağlar Irmak, Elif Işık, Özgür Appak, Muammer Çelik, Gamze Öztürk, Sema Alp Çavuş, Arzu Sayiner, Alp Ergör, Yücel Demiral, Bulent Kilic

**Affiliations:** ^1^ Department of Public Health, Faculty of Medicine Dokuz Eylul University Izmir Turkey; ^2^ Infectious Diseases Unit Diyarbakır Provincial Health Directorate Diyarbakir Turkey; ^3^ Medical Microbiology Unit Mardin Nusaybin State Hospital Mardin Turkey; ^4^ Infectious Diseases and Clinical Microbiology Unit Hakkari Yüksekova State Hospital Hakkari Turkey; ^5^ Department of Medical Microbiology, Faculty of Medicine Dokuz Eylul University Izmir Turkey; ^6^ Department of Infectious Diseases and Clinical Microbiology, Faculty of Medicine Dokuz Eylul University Izmir Turkey

**Keywords:** antibody response, booster doses, COVID‐19, healthcare workers, vaccine

## Abstract

**Background:**

This study is aimed at providing information about the timing of booster doses and antibody kinetics in healthcare workers.

**Methods:**

This research extends a prospective cohort study conducted at Dokuz Eylul University Hospital in Turkey, covering the period from March 2021 to December 2021. During this timeframe, the antibody levels of the health workers were measured at four different time points. The associations of antibody levels with gender, age, occupation, body mass index (BMI), chronic disease, and smoking were analyzed.

**Results:**

There was a significant difference between antibody levels in all four blood draws (*p* < 0.001). Antibody levels decreased in both those vaccinated with BNT162b2 (*p* < 0.001) and those vaccinated with CoronaVac (*p* = 0.002) until the fourth blood draw. There was a significant difference between those vaccinated with one and two doses of booster BNT162b2 before the third blood draw (*p* < 0.001), which continued at the fourth blood draw (*p* < 0.001). The antibody levels of those with an interval of 41–50 days between two vaccinations decreased significantly at the fourth blood draw (*p* < 0.001).

**Conclusions:**

This study provides insight into the dynamics and persistence of antibody response after additional COVID‐19 vaccine doses among healthcare workers. The longer the interval between booster doses may result in greater antibody levels being maintained over time, allowing for longer durations of protection.

## 1 Introduction

Global efforts to combat the coronavirus disease 2019 (COVID‐19) pandemic have triggered a sustained analysis of strategies aimed at optimizing vaccination effectiveness. Within this range of strategies, emphasis has been placed on the administration of booster doses, especially to increase immunity [1]. A booster dose can be defined as an additional vaccine dose administered following the completion of the primary vaccine series [2]. Furthermore, the emergence of severe acute respiratory syndrome coronavirus 2 (SARS‐CoV‐2) variants sustained by a decrease in immunity after infection or vaccination highlights the crucial need for additional doses of COVID‐19 vaccines [3]. In a retrospective study conducted in the United States, it was revealed that vaccine effectiveness against infection decreased to approximately 50% 7 months after the third dose [4]. In a systematic review and meta‐analysis conducted with the four vaccines with the most data (BNT162b2, mRNA 1273, Ad26.COV2.S, and ChAdOx1‐S), it was stated that vaccine effectiveness decreased by 8% in a 6‐month analysis in all age groups [5]. Based on existing evidence indicating that protective immunity decreases within 4–6 months of first vaccination [6, 7], it may be advisable to receive a booster dose if possible. Among healthy adults, receiving a booster dose following a primary immunization series has been proven to significantly increase immunogenicity and improve peak antibody levels [8].

As new variants emerged and immunity of the individuals diminishes, the third and fourth vaccines were administered in some countries. With these booster doses, protection against infection was increased and defense against severe COVID‐19 was increased [9]. The third dose yields an increase in both humoral and cellular immunity, regardless of the type of initial doses and the type of booster dose itself [10]. In the study of Bar‐On et al., it was stated that the fourth dose provided additional protection against infections and serious disease caused by the Omicron variant, compared to the third booster dose administered 4 months ago [11]. In the study of Li et al., it was stated that a booster dose rapidly triggered strong immune responses in patients who received 2 doses of CoronaVac [12].

However, these booster vaccine applications not only increase costs but also take time. Therefore, it is important to determine the timing of booster vaccinations [9]. Park et al. showed that while vaccination is recommended every 6–12 months to prevent severe COVID‐19 in the elderly and immunocompromised patients, frequently repeated vaccinations would be of limited benefit in the young population [12]. While adults aged 50 and over were considered at risk in September 2021, it was recommended to apply booster doses to cover all adults in November 2021. The booster dose was initially advised 6 months after the completion of the first vaccine doses, although this interval was shortened to 3 months with arrival of Omicron variant [13]. In countries with early booster dose campaigns such as Israel, the United Kingdom, and the United States, numerous people, notably those who are more vulnerable to severe COVID‐19, had received their booster dose months before the onset of the Omicron wave. Policymakers have contemplated providing a fourth vaccine dose to the most vulnerable as a possible defense against the Omicron variant [14]. On January 3, 2022, the Israeli Ministry of Health launched a fourth vaccination campaign for high‐risk individuals, 4 months after the third vaccination [15]. In the United States, in response to the Omicron wave, the period between the third and the fourth doses for immunocompromised individuals was reduced from 6 to 5 months [16]. Vaccination campaigns for the fourth dose were carried out in countries like the United Kingdom [17]. In Turkey, the third dose of the COVID‐19 vaccine was recommended by the Ministry of Health to people who have received two doses of inactive vaccine at least 3 months after the last dose [18]. Despite studies on prioritization of the use of the COVID‐19 vaccine [19, 20], information on the timing of COVID‐19 booster vaccination is not sufficient [12]. However, given the variety of COVID‐19 diseases in the population, the efficiency of COVID‐19 booster doses at different frequencies may vary according to significant risk factors [12].

This enhanced focus is especially crucial for at‐risk populations, such as healthcare workers, who are frequently exposed to the risk of infection [21]. In addition to being at the forefront of combating the pandemic, healthcare workers play an essential role in maintaining the functionality of healthcare systems [22] and are constantly exposed to potential infection [21]. Therefore, investigating the effect of additional vaccine doses on the immune response becomes critical for informed public health decisions. Understanding antibody kinetics after the third and fourth vaccine doses is vital to elucidate the optimal vaccination strategy, sustainable protection against severe outcomes, and ultimately contribute to the global effort to control the spread of the virus. This may influence vaccine guidelines, particularly for healthcare workers who play a key role in the ongoing fight against COVID‐19, by providing a basis for evidence‐based decision‐making in public health initiatives. This study is aimed at providing information about the timing of booster doses and at generating an understanding on the waning of humoral immunity against time and booster doses in healthcare workers.

## 2 Methods

### 2.1 Study Design and Population

This study is the continuation of the prospective cohort research conducted at Dokuz Eylul University Hospital in Turkey, the first results of which were published [23]. Dokuz Eylul University Hospital is a tertiary hospital. The hospital has a capacity of 1200 beds. There are 50,000 patient admissions to the hospital annually.

During the COVID‐19 period, the flow of information was rapid, there was a lack of experience with vaccines, and Turkey was one of the rare countries where heterologous booster application was performed after primary vaccination with CoronaVac. Briefly, in our first article, we evaluated the anti‐receptor‐binding domain (RBD) immunoglobulin G (IgG) levels after two doses of CoronaVac at first month (median: 469.2 AU/mL) and fourth month (median: 166.5 AU/mL) and within 2 months after the third dose of CoronaVac or BNT162b2 among 560 uninfected healthcare workers. The antibody level increased 104.8‐fold (median: 17609.4 vs. 168 AU/mL) and 8.7‐fold (median: 1237.9 vs. 141.4 AU/mL) in the participants who received BNT162b2 and CoronaVac, respectively.

In this study, we evaluated the antibody levels after third and fourth dose vaccination within the follow‐up period between July 1, 2021, and December 15, 2021.

### 2.2 Data Collection and Monitoring of Antibody Levels

**FIGURE 1:**
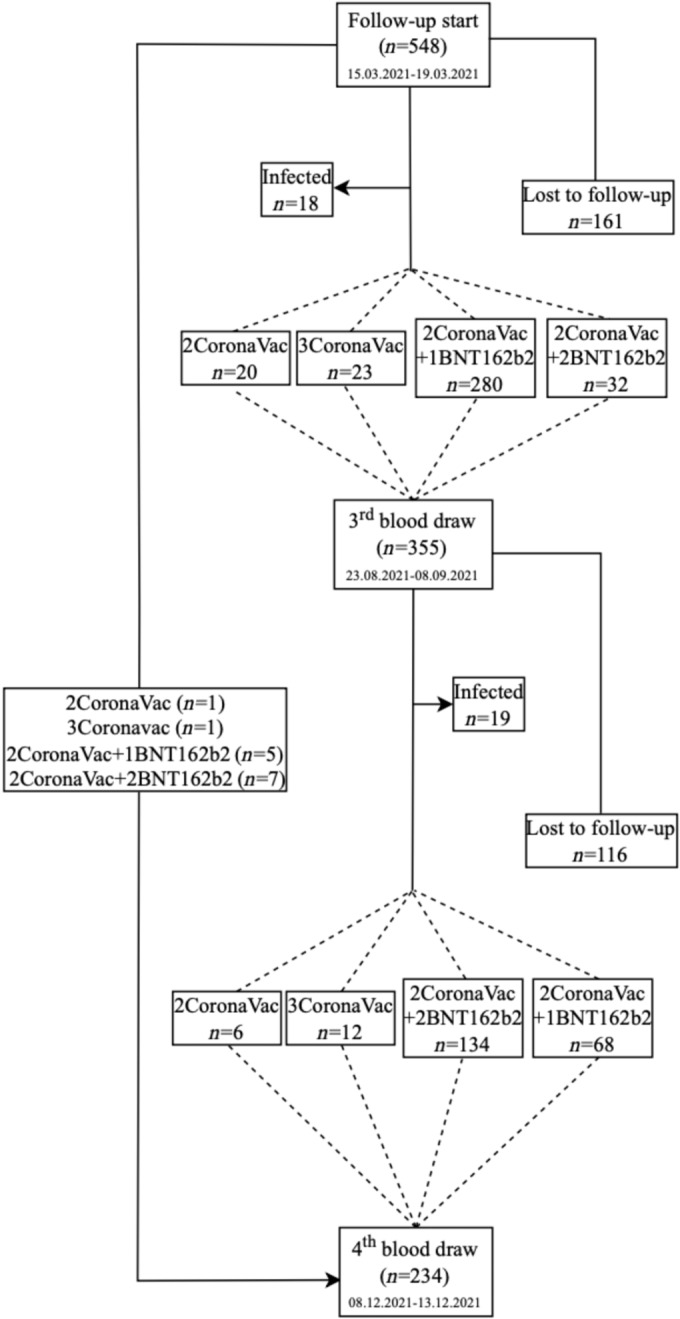
Study population flowchart.

On July 1, 2021, a third dose reminder vaccination schedule (CoronaVac or BNT162b2 depending on individual preference) was defined for healthcare workers. On August 16, 2021, a fourth dose was defined for those traveling to countries that require two doses of BNT162b2. Between August 23 and September 3, 2021, third blood samples, and December 8–13, 2021, fourth blood samples were collected to determine the antibody levels. The outcome variable of the study was quantitative antibody level. Independent variables are gender, age, occupation, body mass index (BMI), chronic disease status, and smoking. SARS‐CoV‐2 infection in the participants was regularly monitored in the Occupational Health and Safety Unit of the hospital. Healthcare workers who were found to be positive by PCR test and healthcare workers who were vaccinated with a booster dose up to 7 days before blood draws were not included in the analysis. The current vaccination status of healthcare workers before blood draw, those who were lost to follow‐up from the study, and healthcare workers who were found to be infected before blood drawn is shown in Figure 1.

### 2.3 Laboratory Assay

Serum samples were tested for anti‐RBD‐IgG antibody with a SARS‐CoV‐2 IgG II QUANT (Abbott Diagnostics) assay on the Architect analyzer. The quantitation range of the test was 50–40,000 AU/mL.

### 2.4 Statistical Analysis

**FIGURE 2:**
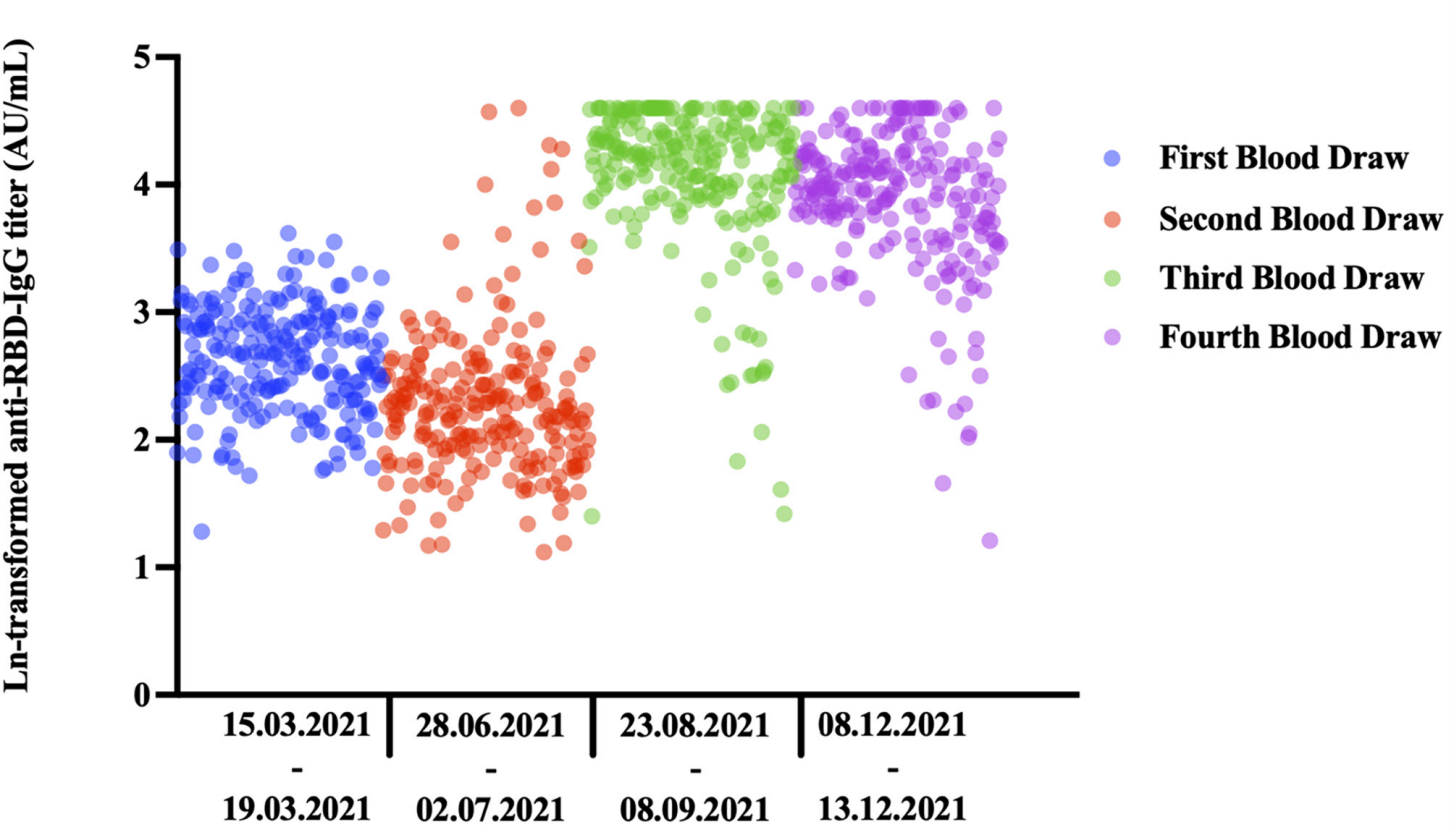
Comparison of antibody levels of healthcare workers according to blood draw time. Due to the extreme values of the antibody level in the graphs, the natural logarithm is shown. IgG, immunoglobulin G; RBD, receptor‐binding domain.

**FIGURE 3:**
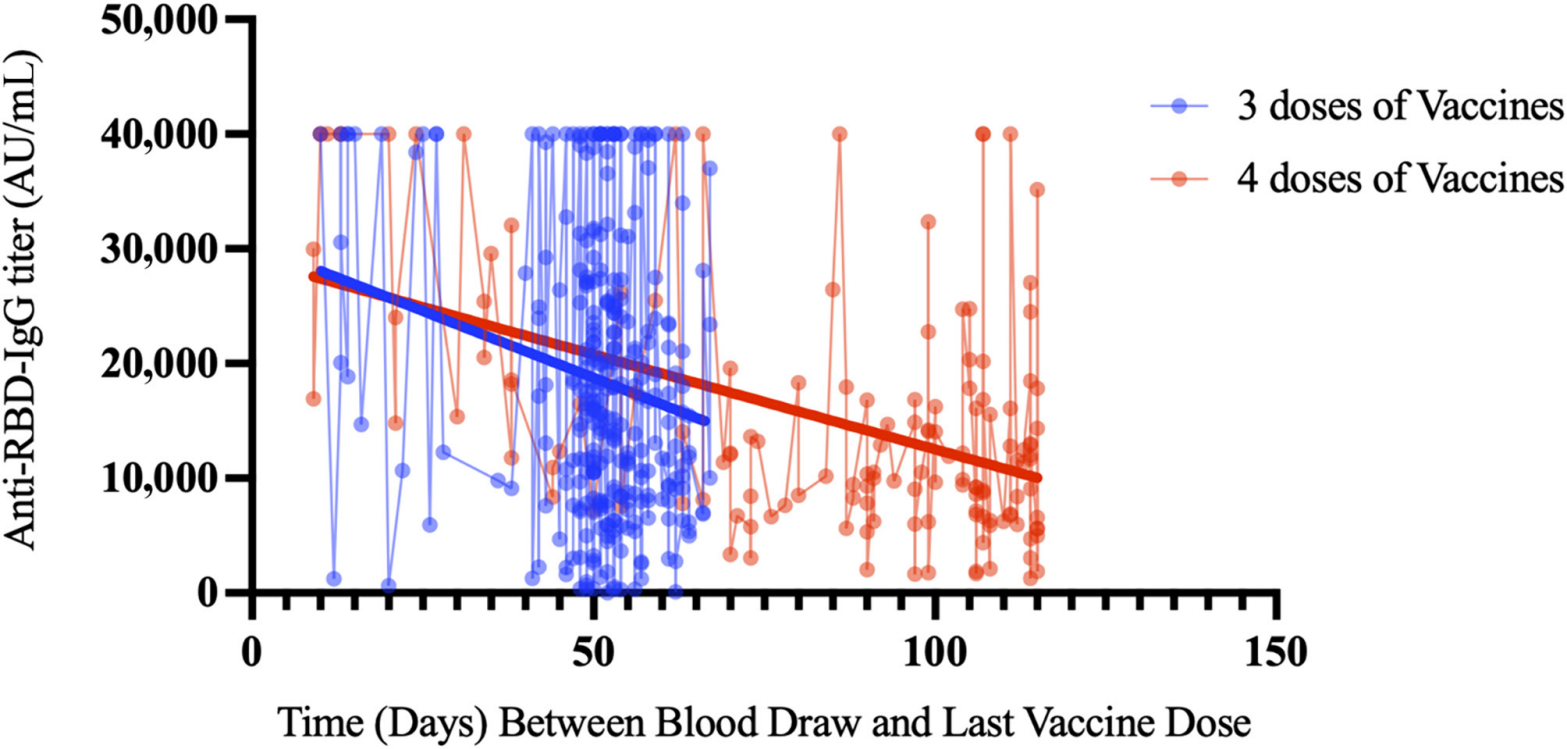
Monitoring anti‐RBD‐IgG titer (arbitrary unit per milliliter) levels in healthcare workers vaccinated with two CoronaVac and one and two doses of BNT162b2.

Descriptive statistics were generated in numbers and percentages for categorical variables. Conformity of continuous variables to normal distribution was examined visually (by means of histograms and probability graphs) and statistically (by means of Kolmogorov–Smirnov/Shapiro–Wilk tests). Continuous variables which do not fit the normal distribution, the median, and 25th–75th percentile (interquartile range) were used. Categorical variables were compared using Pearson *χ* ^2^ or Fisher's exact tests. Since quantitative antibody levels did not show a normal distribution for the sociodemographic variables compared, the Mann–Whitney *U* test was used for two groups and the Kruskal–Wallis test was used for more than two groups. The Bonferroni correction was used when the result was statistically significant in the Kruskal–Wallis test by employing a pairwise comparison of groups. The Wilcoxon test was used for quantitative antibody level comparison in dependent groups. Analyses were performed using SPSS 26.0 (IBM Corporation), and *p* < 0.05 was considered statistically significant. Flowchart was prepared in draw.io application. Figures 2 and 3 are prepared in the GraphPad Prism 10.

## 3 Results

Until the third blood draw, 280 healthcare workers received 1 booster dose of BNT162b2 (10–67 days, median: 52 days), 23 healthcare workers received 1 booster dose of CoronaVac (12–62 days, median: 49 days), and 32 healthcare workers received 2 booster doses of BNT162b2 (8–20 days, median: 10.5 days). Until the fourth blood draw, 73 healthcare workers received 1 booster dose of BNT162b2 (84–161 days, median: 156 days), 13 healthcare workers received 1 booster dose of CoronaVac (121–161 days, median: 155 days), and 141 healthcare workers received 2 booster doses of BNT162b2 (9–115 days, median: 97 days).

While significantly higher antibody levels were observed in men than in women at the third blood draw (*p* = 0.027), there was no significant difference between genders at the fourth blood draw (*p* = 0.343). There was no significant difference between age groups in the third blood draw (*p* = 0.129) and the fourth blood draw (*p* = 0.722). At the third blood draw, medical doctors had considerably higher antibody levels than nurses and other healthcare workers (*p* = 0.020); although at the fourth blood draw, there was no difference in antibody levels by profession (*p* = 0.594). While healthcare workers who were overweight according to their BMI had significantly lower antibody levels than those who were obese and normal‐thin (*p* = 0.023), there was no significant difference between the groups for BMI at the fourth blood draw (*p* = 0.611). Antibody levels in both the third (*p* = 0.217) and fourth blood draws (*p* = 0.843) of healthcare workers with and without chronic disease were not significantly different. Antibody levels were not significantly different according to smoking status at both the third (*p* = 0.745) and fourth blood draw (*p* = 0.766) (Table 1).

**TABLE 1:** Comparison of antibody levels in third and fourth blood draws according to selected variables.

	*n* (%)	Third blood draw antibody level (three doses vaccinated) AU/mL median (IQR)	*p*	*n* (%)	Fourth blood draw antibody level (four doses vaccinated) AU/mL median (IQR)	*p*
Sex						
Women	222 (73.7)	15,550.5 (17,361.9)	**0.027**	104 (74.8)	11,439.5 (11,737.2)	0.343
Men	79 (26.3)	18,392.4 (27,556.5)		35 (25.2)	13,218.0 (8868.0)	
Age groups						
20–35	97 (32.2)	18,143.3 (19,857.7)	0.129	33 (25.4)	13,006.0 (11,055.4)	0.722
36–50	149 (49.5)	15,416.7 (19,139.7)		67 (51.5)	11,368.8 (10,293.6)	
51–65	55 (18.3)	13,892.8 (15,353.0)		39 (23.1)	11,351.2 (17,449.8)	
Occupation						
Medical doctor	100 (33.2)	21,451.1 (24,961.7)	**0.020**	47 (36.2)	12,906.5 (11,965.0)	0.594
Nurse	58 (19.3)	14,906.5 (14,145.1)		24 (18.5)	9523.1 (9273.3)	
Other	143 (47.5)	14,680.2 (17,481.7)		68 (52.3)	12,119.0 (11,177.5)	
Body mass index						
Normal‐underweight	138 (45.8)	17,751.2 (17,965.5)	**0.023**	59 (42.4)	12,333.8 (12,725.4)	0.611
Overweight	113 (37.5)	13,946.3 (15,928.5)		56 (40.3)	10,723.8 (11,212.2)	
Obese	50 (16.6)	18,088.1 (30,916.8)		24 (17.3)	12,119.0 (14,802.6)	
Chronic disease						
No	225 (74.8)	17,086.4 (17,744.3)	0.217	95 (68.3)	11,984.0 (10,696.8)	0.843
Yes	76 (25.2)	14,668.7 (21,061.5)		44 (33.7)	11,541.4 (12,688.0)	
Smoking						
Never	173 (57.5)	16,511.1 (18,343.5)	0.745	79 (56.8)	12,333.8 (10,997.4)	0.766
Current	79 (26.2)	14,739.2 (19,184.2)		27 (19.4)	9643.0 (14,626.2)	
Ever	49 (16.2)	17,086.4 (15,168.3)		33 (23.7)	11,386.8 (9042.7)	

*Note:* Values in bold are represent a significant difference.

All blood samples taken within the scope of the project were shown in the figure. Third and fourth blood draws were included in this study. There was a significant difference between antibody levels in all blood draws (Friedman: *p* < 0.001). When blood samples were compared in groups of two, there was a significant difference in terms of quantitative antibody levels in all analyses (applicable for all analyses, Wilcoxon: *p* < 0.001, 1 > 2, 1 < 3, 1 < 4, 2 < 3, 2 < 4, and 3 > 4) (Figure 2). In healthcare workers vaccinated with two doses of CoronaVac, even a single booster dose of BNT162b2 resulted in peak levels of anti‐RBD‐IgG titers. This effect continued with a small decrease after the second booster dose.

The median antibody level in 20 healthcare workers vaccinated with 2 doses of CoronaVac at the third blood draw was 231.6 (500.3), and the median antibody level in 7 healthcare workers vaccinated with 2 doses of CoronaVac at the fourth blood draw was 206.3 (4805.9). There was no significant decrease in antibody levels between two blood draws (*p* = 0.345) (Table 2).

**TABLE 2:** Evaluation of antibody levels following varied COVID‐19 vaccination schedules.

		Third blood draw antibody level (*n* = 355)	Fourth blood draw antibody level (*n* = 234)	*p* value*
		AU/mL median (IQR)	AU/mL median (IQR)	
2 doses vaccinated				
CoronaVac		231.6 (500.3) (*n* = 20)	206.3 (4805.9) (*n* = 7)	0.345
3 doses vaccinated				
BNT162b2 CoronaVac		17,751.2 (17,107.4) (*n* = 280) 1277.6 (2202.9) (*n* = 23)	5620.2 (7765.1) (*n* = 73) 323.4 (1554.4) (*n* = 13)	< 0.001 0.002
4 doses vaccinated				
BNT162b2		39,125.8 (13,674.2) (*n* = 32)	11,984.0 (11,022.0) (*n* = 141)	< 0.001
		< 0.001 (*p* value**)	< 0.001 (*p* value**)	*p* value*
4 doses vaccinated				
BNT162b2 (intervals of 41–50 days)	39,125.8 (13,674.2) (n = 32)	11,369.0 (7982.9) (*n* = 38)	< 0.001	
4 doses vaccinated	—	11,849.5 (10,503.0) (*n* = 90)	—	
BNT162b2 (intervals of 51–134 days)				
4 doses vaccinated	—	40,000.0 (15,981.9) (*n* = 11)	—	
BNT162b2 (intervals of 135–150 days)				
		< 0.001 (*p* value***)		

*Note:* The vaccine groups above are presented according to vaccination status before blood draw.

* The Wilcoxon test was used for quantitative antibody level comparison in dependent groups.

** Mann Whitney *U* test was used for one dose of BNT162b2 booster dose vs. two doses of BNT162b2.

*** Kruskal–Wallis *H* test was used (who received two booster doses of BNT162b2 at the 2nd blood draw, grouped by interval between Doses 3 and 4).

Among healthcare workers vaccinated with 3 doses before the third blood draw, the median antibody level of 280 participants vaccinated with 1 booster dose of BNT162b2 was 17,751.2 (17107.4) AU/mL, while the median antibody level of 23 participants vaccinated with 1 booster dose of CoronaVac was 1277.6 (2202.9) AU/mL. The median antibody level of 73 participants vaccinated with 1 booster dose of BNT162b2 until the fourth blood draw was 5620.2 (7765.1) AU/mL, and the median antibody level of 13 participants vaccinated with 1 booster dose of CoronaVac was 323.4 (1554.4) AU/mL. Antibody levels decreased in both those vaccinated with BNT162b2 (*p* < 0.001) and those vaccinated with CoronaVac (*p* = 0.002) until the fourth blood draw. The median antibody level of 32 participants in healthcare workers vaccinated with 4 doses until the third blood draw was 39,125.8 (13674.2) AU/mL, and the median antibody level in healthcare workers vaccinated with 4 doses until the fourth blood draw was 11,984.0 (11022.0) AU/mL. Antibody levels at the fourth blood draw were significantly lower than those at the third blood draw (*p* < 0.001). There was a significant difference between those who vaccinated with one and two doses of booster BNT162b2 before the third blood draw (*p* < 0.001), which continued at the fourth blood draw (*p* < 0.001) (Table 2).

In healthcare workers who received 4 doses of vaccine and had an interval of 41–50 days between 2 booster doses, the median antibody level of the third blood draw was 39,125.8 (13674.2) AU/mL, and the median antibody level of the fourth blood draw was 11,369.0 (7982.9) AU/mL. For those who have 51–134 days between 2 booster doses of vaccine, the median antibody level of the fourth blood draw was 11,849.5 (10,503.0) AU/mL. For those who have 135–150 days between 2 booster doses, the median antibody level of the fourth blood draw was 40,000.0 (15,981.9) AU/mL, while the antibody levels of those with an interval of 41–50 days between 2 booster doses decreased significantly at the fourth blood draw (*p* < 0.001). Mann Whitney *U* test was used to compare who received 2 booster doses of BNT162b2 at the fourth blood draw, grouped by time between Doses 3 and 4 (intervals of 41–50 vs. intervals of 51–134 days, *p* = 0.071; intervals of 41–50 vs. intervals of 135–150 days, *p* < 0.001; and intervals of 51–134 vs. intervals of 135–150 days, *p* < 0.001). Mann Whitney *U* test was used to compare who received 2 booster doses of BNT162b2 at the fourth blood draw, grouped by time between Doses 3 and 4 (1 booster dose BNT162b2 vs. intervals of 51–134 days, *p* = 0.015; 1 booster dose BNT162b2 vs. intervals of 135–150 days, *p* < 0.001; and 1 booster dose BNT162b2 vs. intervals of 135–150 days, *p* < 0.001) (Table 2).

Anti‐RBD‐IgG titer (arbitrary unit per milliliter) levels showed a similar change in healthcare workers vaccinated with one and two booster doses of BNT162b2 from blood draw to the last dose of vaccination. Anti‐RBD‐IgG titer (arbitrary unit per milliliter) levels could be monitored during longer follow‐up periods in healthcare workers vaccinated with two booster doses of BNT162b2 (Figure 3).

## 4 Discussion

In our study, antibody levels decreased in a period of 3–4 months. As the number of BNT162b2 booster doses increases, healthcare workers' anti‐RBD‐IgG antibody levels increase. Booster doses administered close to the blood draw increase anti‐RBD‐IgG antibody levels to peak values. In our study, the superiority of BNT162b2 in terms of antibody response to CoronaVac is observed. Blood anti‐RBD‐IgG antibody levels have been found to be high in men, doctors, and overweight people.

In the study by Kim et al., it was stated that in healthcare workers vaccinated with booster BNT162b2, S1‐IgG antibody levels decreased to levels at which protection against infection would not occur after 6.5 months [24]. In the study of Desmecht, Tashkeev, and El Moussaoui, the benefit of the booster dose was greater in susceptible individuals. Personalization of mRNA vaccination protocols used to prevent severe COVID‐19 and reduce the impact of the epidemic on the healthcare system is gaining importance [25]. Tanir et al. evaluated the anti‐S‐RBD‐IgG levels among vaccine dose subgroups at 1, 3, 6, and 12 months, regardless of the vaccine type. At the end of the first year, antibody levels were higher in those who received four or five doses versus those who received two or three doses. This study advised a vaccination regimen of 0, 1, 5, and 9 months for high‐risk populations such the elderly, healthcare professionals, and persons with impaired immune systems [26]. The study of Jung et al. included the recommendation to administer additional booster doses at least 6–12 months after the third dose of vaccination. In this study, it was found that the decrease in immunity seen after one booster dose of BNT162b2 vaccination showed a similar pattern to that of two doses of BNT162b2 vaccination [27].

A similar decrease in our study is shown in Figure 3. It appears that this effect seen on patients is dose dependent and additional doses are needed as vaccine effectiveness decreases over time. In our study, it was observed that antibody levels decreased over a period of 3–4 months. In healthcare workers vaccinated with two booster doses of BNT162b2 after two doses of CoronaVac vaccine, antibody levels were measured at peak levels when the time from vaccination to blood collection was short. However, in our study, it was observed that in cases where the period between vaccination and blood draw was longer than 15 days, keeping the interval between two booster doses close or widening the interval did not cause a difference in terms of measured antibody levels. This suggests that spacing the booster doses is the strategy that should be applied for a more appropriate immunization. Various studies have highlighted the fact that a long vaccination interval is suitable for strong humoral immunogenicity [28, 29, 30]. However, Voutouri et al. found that antibody levels above 10,000 U/mL for 70–80 weeks and over 1000 U/mL for 180 weeks could be observed in patients receiving a single dose of mRNA vaccine [31]. In the study of Falsey, Frenck, and Walsh, it was stated that leaving a period of 6 months between BNT162b2 booster vaccinations after two 3‐week BNT162b2 intervals would result in stronger immune responses [32].

In our study, healthcare workers who received one booster dose of BNT162b2 had significantly lower anti‐RBD‐IgG antibody levels than those who received two booster doses of BNT162b2 at the third blood draw. This difference was consistent at the fourth blood draw. Nevertheless, for each time interval compared, two doses of BNT162b2 were shown to be superior to one dose of BNT162b2. Nevertheless, our study could not completely support the assumption that two booster doses were better than one booster dose since blood was obtained from healthcare workers who had received two booster doses more recently. Voutouri et al. found that the time interval between receiving the fourth booster dose had no significant effect on cellular immunity or viral infection severity. It was found that cell immunity and severity of infection were not strongly affected regardless of when the second booster dose was given, 3–6 months after the first booster dose [31]. In addition, in the study conducted by Parry et al. in elderly participants, it was shown that long‐interval vaccination increased the peak antibody response by 3.5 times compared to the standard vaccination interval [33]. A similar effect was clearly seen in the third and fourth blood draws of healthcare workers who recently received two doses of the BNT162b2 booster vaccine. Garg et al. found that a delayed booster dose improved antibody response by increasing affinity maturation [34]. Payne et al. found that antibody levels were higher after the extended regimen than after the short regimen. In the study, it was stated that a longer dose interval caused an increase in peak neutralizing antibody levels and B cells but did not lead to an increase in T cells [35]. Therefore, in addition to the effect on humoral immunity seen in our study, the effect on cellular immunity may also need to be seen in future studies.

In our study, it was found that two booster doses of BNT162b2 vaccine administered recently at both blood collection times increased antibody levels to a very high level. Munro et al. found that the maximal responses obtained after the fourth dose may be similar or better than the third dose [36]. Eliakim‐Raz et al. found that IgG titers increased after the third dose and then decreased nearly tenfold after 5 months. Furthermore, antibody titers were increased again immediately following the fourth dose [37]. This supports the effect of recent booster doses on antibody responses. Furthermore, it has been shown that the optimum immunogenicity of mRNA vaccines was attained after three doses and can only be maintained by the fourth dose [38]. Although a similar decrease was observed in the third and fourth blood draws in those vaccinated with three and four doses of BNT162b2 booster dose, the decreasing trend was less in healthcare workers vaccinated with one more dose of BNT162b2 booster dose.

In our study, the superiority of BNT162b2 over CoronaVac in terms of increasing antibody levels is consistent with the literature. In healthcare workers vaccinated with three doses, vaccination with BNT162b2 is superior to vaccination with CoronaVac in both blood measurements. The weaker and shorter term immune responses observed after the CoronaVac vaccine in Cowling et al.’s study support the need to administer the third dose at an earlier stage for individuals who previously received two doses [39]. This could be an important consideration for revising the vaccination schedule to strengthen vaccine‐induced immune responses. Niyomnaitham et al. found that a booster dose of BNT162b2 could improve the immunogenicity of homologous CoronaVac primary frames but was unlikely to protect against Omicron, requiring a third booster dose [40]. Çulpan, Aydın, and Uygur found that one or two doses of the BNT162b2 booster showed higher efficacy compared to a single dose of the CoronaVac booster [41]. In the study by Costa Clemens et al., the most significant increases in antibody levels were seen when a booster dose of mRNA vaccine was administered, mostly to participants previously vaccinated with two doses of the CoronaVac vaccine [42]. It is still unclear why standard and extended interval vaccinations show different responses. However, mRNA vaccines specifically stimulate antibody responses strongly [43], although their effects on cellular immunity are not fully resolved [44].

At the third blood draw, quantitative antibody levels were considerably greater in males than in women, in medical doctors compared to other healthcare professionals, and in overweight persons compared to obese and normal‐weight people. Antibody levels at the fourth blood draw were not found to be different for any variable compared. In the study by Tanır et al., anti‐S‐RBD levels measured at the 12th month were found to be higher in women, healthcare workers who were vaccinated more frequently, those who received heterologous vaccines, and those who were vaccinated with BNT162b2 [26]. In the study by Cowling et al., stronger and more persistent antibody levels were observed against two doses of the mRNA vaccine in young people and women [39]. Levin et al. found that the immune response was suppressed and the humoral response was considerably decreased in men and those over the age of 65 who were vaccinated with two doses of BNT162b2, 6 months after the second dose [45]. In the study by Desmecht, Tashkeev, and El Moussaoui, a significant relationship was observed between age and the persistence of the humoral response [25]. Choi et al. found no association between age, BMI, comorbidities, and antibody levels [46]. The gender difference found in our study may be due to the nature of humoral immunity, and the difference in terms of professions may be since doctors are a younger group. Furthermore, the fact that most nurses are women may have influenced this discrepancy. Since antibody levels increased to a ceiling level with an additional dose at the fourth blood draw, we may not have found any differences for the variables in the previous analysis.

### 4.1 Strengths and Limitations

The cohort included in the study was well monitored for infection since the participants were healthcare workers employed in the same hospital. Since the evaluations revealed that anti‐RBD‐IgG antibody levels were high in positive healthcare workers, COVID‐19 positive patients were excluded from some analyses to avoid selection bias. All participants in the study received two doses of the CoronaVac vaccine in the same period, but the third and fourth doses were left to the participants' preference, making it difficult to compare antibody levels at the third and fourth blood draws. For this reason, no comparison could be made in terms of homologous vaccination in our study. Between the third and fourth blood draw, 34% of the participants were lost to follow‐up. Although this was random, the decrease in the number of participants resulted in small sample size for comparison among groups. Despite the limitations, it is one of the few studies which shows the antibody response in participants who received m‐RNA vaccines as one or more booster doses after the primary immunization with CoronaVac. Our study was conducted among healthcare professionals at a university hospital. Therefore, the generalizability of the findings is limited. Due to the specific characteristics of university hospital staff, results may not be representative of broader populations or other healthcare settings. Additional research with different populations is needed to confirm our findings.

## 5 Conclusions

This study provides insight into the dynamics and persistence of antibody response after additional COVID‐19 vaccine doses among healthcare workers. The findings emphasize the necessity of understanding trends over time and vaccine type concerns for developing immunization strategy for this high‐risk group. Spacing between booster doses may result in stronger antibody levels being maintained over time, allowing for extended periods of protection. Groups at high risk of serious disease and healthcare workers should be targeted. This study provides another piece of evidence for making decisions on the timing of booster doses. These findings need to be supported by studies with larger numbers of participants, as well as taking the variants into account.

## Author Contributions

**Ahmet Furkan Süner:** conceptualization, investigation, writing – original draft, writing – review and editing, visualization, methodology, project administration, formal analysis, data curation, resources, validation. **Gül Ergör:** conceptualization, methodology, validation, data curation, supervision, investigation, writing – original draft, writing – review and editing, visualization, project administration. **Derya Çağlayan:** methodology, validation, supervision, project administration, conceptualization, investigation, writing – original draft. **Neslişah Türe:** project administration, conceptualization, investigation. **Irmak Güzel:** project administration, conceptualization, investigation. **Çağlar Irmak:** conceptualization, investigation. **Elif Işık:** conceptualization, investigation. **Özgür Appak:** formal analysis, project administration, supervision, investigation, conceptualization. **Muammer Çelik:** conceptualization, investigation, supervision, writing – review and editing. **Gamze Öztürk:** conceptualization, investigation. **Sema Alp Çavuş:** investigation, conceptualization, supervision, project administration. **Arzu Sayiner:** supervision, project administration, methodology, validation, conceptualization, investigation, writing – review and editing. **Alp Ergör:** methodology, supervision, project administration, conceptualization, investigation. **Yücel Demiral:** methodology, writing – review and editing, supervision, project administration. **Bulent Kilic:** supervision, project administration, conceptualization, methodology.

## Ethics Statement

The study was approved by the Dokuz Eylul University Clinical Research Ethics Committee (No. 2021/07‐01), Turkish Medicines and Medical Devices Agency Clinical Research Department (code: 21‐AKD‐33), and TR Ministry of Health General Directorate of Health Services (2021‐02‐12T15_05_18).

## Conflicts of Interest

The authors declare no conflicts of interest.

## Data Availability

The data that support the findings of this study are available on request from the corresponding author. The data are not publicly available due to privacy or ethical restrictions.
